# Succinylation – encoded metabolic codes: cracking the molecular logic of cellular adaptation

**DOI:** 10.1097/JS9.0000000000003249

**Published:** 2025-08-26

**Authors:** Yipeng Cong, Xiaoman Zhang, Zian Wang, Zhongren Cui, Chengming Li, Yongzheng Han, Wen Deng, Xingxuan Zhou, Hongliang Wu, Jingsong Sun, Hongbo Fan, Guangzhen Wu

**Affiliations:** a Department of Urology, The First Affiliated Hospital of Dalian Medical University, Dalian, China; b Department of Neurology Intensive Care Unit, The Second Affiliated Hospital of Dalian Medical University, Dalian, China; c Department of Orthopedics, The First Affiliated Hospital of Dalian Medical University, Dalian, China

**Keywords:** dessuccinylase tricarboxylic acid cycle, immune escape, succinylation, tumorigenesis, tumor microenvironment

## Abstract

Succinylation, a recently recognized post-translational modification of lysine, has been found to be of crucial importance in the metabolic reprogramming of tumors. Succinylation plays a significant role in determining the metabolic state of cells by regulating the activities and stability of key enzymes in metabolic pathways such as the tricarboxylic acid (TCA) cycle, oxidative phosphorylation, fatty acid metabolism, and glycolysis. In addition to that, succinylation regulates immune checkpoint molecules (e.g., PD-L1, CD47), antigen presentation, and tumor immune microenvironment homeostasis. Recent studies illustrate that desuccinylase (e.g., SIRT5 and SIRT7) and succinyltransferase (e.g., KAT2A, CPT1A, HAT1, and alpha-KGDH) expression and malfunction are strongly related to immune escape of cancer. Accordingly, it has the potential for adjuvant drug targets after surgical treatment. Integrating small-molecule agents that modulate succinylation-related enzymes with surgical intervention represents a novel therapeutic approach aimed at decreasing tumor recurrence following surgery, augmenting immunotherapeutic responsiveness, and ultimately improving patients’ long-term survival outcomes. This review comprehensively examines the regulatory roles of succinylation in the tumor microenvironment and recent advances in oncological therapeutics, providing a theoretical foundation for integrated post-surgical cancer care and targeted therapy design, and identifying potential clinical oncology targets.

**Graphical abstract:**

http://links.lww.com/JS9/G282

## Introduction

### Modification of proteins

Protein modification, known as post-translational modification (PTM), involves chemical changes that occur in proteins after synthesis, playing a crucial role in regulating protein degradation and maintaining cellular homeostasis. Different types of modifications, their combinations, and modification site variations can potentially lead to enormous variations in protein properties and functions, thereby influencing a wide range of cellular processes[1]. Lysine is a required amino acid which is accountable for protein structure as well as function, and it is one of the three primary residues which carry out these tasks. Lysine side chain modification is the single biggest contributor to PTM network complexity. Among amino acids that constitute proteins, lysine is most likely a modified residue post-translation[2]. The lysine residues of proteins are subject to a variety of PTMs such as methylation, acetylation, biotinylation, ubiquitination, ubiquitin-like modifications, propionylation, and butyylation^[3,4]^. Succinylation is a new and recently discovered PTM[2]. By transferring succinyl groups (–CO–CH2–CH2–CO2H) to specific protein residues, succinylation modulates protein structure and function, thereby participating in diverse cellular processes critical to human biology[5] and affects the development of many diseases.

Extensive evidence supports the role of succinylation regulators in modulating the progression of multiple cancer types, including gastric, colon, lung adenocarcinoma, breast, pancreatic, esophageal squamous cell carcinoma, and osteosarcoma. These regulators exert their effects by modulating the succinylation levels of specific substrate targets[5], thereby influencing the development of diverse cancer types.

### Metabolic reprogramming

Metabolic reprogramming is a hallmark of cancer, providing cancer cells with the necessary energy and metabolic intermediates to fuel tumor growth and evade apoptosis[6]. Post-translational modification is also one of the crucial steps in metabolic reprogramming, including increased glycolysis, decreased tricarboxylic acid (TCA) activity, increased fatty acid synthesis, inflammation response, and amino acid metabolism in the urea cycle. Protein succinylation is closely linked to cancer metabolism and is important to metabolic remodeling of biological cells. Since succinyl-CoA is synthesized in mitochondria, succinylation is a natural biological process that modulates key metabolic processes in mitochondria, such as TCA cycle, fatty acid metabolism, and ATP production. This modification impacts cellular glucose, amino acid, and lipid metabolism by reshaping the structure and function of proteins, thereby influencing both physiological and pathological cellular processes[7], ultimately contributing to tumorigenesis.

### Tumor microenvironment

Tumor microenvironment (TME) is the “milieu” of the tumor cells[8], which plays a very important role in research work in the process of tumor growth, metastasis, dissemination, and therapy response[9]. TME consists of complexly structured components, including neoplastic cells, immunocytes, stromal fibroblasts, and vascular endothelial cells. Depending on the varied contents of TME, it has been proven to be a therapeutic target according to corresponding pathways or active substances[10]. They include immunotherapy, anti-angiogenic therapy, and cancer-associated fibroblasts and extracellular matrix treatment[11], the most efficient and irreplaceable anticancer therapy being immunotherapy[12]. Conversely, TME causes hypoxia and acidity by promoting vascular structure disorder and vascular stiffness, hence triggering tumor invasion and drug resistance[13]. For instance, TME causes dysfunction of immune cells and generation of immunosuppression via PD-1/PD-L1 signal pathways[14]. Comprising both cellular and non-cellular elements, the TME collectively influences tumor progression, immune evasion, metastasis, and resistance to therapy.

In the tumor microenvironment, various conditions like Warburg effect, hypoxia, pH imbalance, and cellular metabolism affect changes in the concentration of the donors of succinylation and the activity of regulators of succinylation, thereby affecting the extent of protein succinylation^[15–20]^ TME serves as the “ecosystem” of tumor cells[8], which is of great value for research on tumor growth, metastasis, spread and response to treatments[9]. TME comprises complex structures, including tumor cells, immune cells, fibroblasts, and vascular endothelial cells. According to the various components of TME, it has been found that it can be targeted as therapy based on associated pathways or active molecules[10]. Examples include immunotherapy, anti-angiogenic agents, and therapies targeting cancer-associated fibroblasts and extracellular matrix[11], out of which immunotherapy is a successful and essential cancer therapy[12]. Nevertheless, TME can induce hypoxia and acidosis due to vascular structure disorder and vascular stiffness, thus promoting tumor invasion and drug resistance[13]. For instance, TME can impair immune cell function and foster immunosuppression via the PD-1/PD-L1 signaling pathways[14]. TME consists of numerous cellular and non-cellular components, together contributing to tumor progression, immune evasion, metastasis, and drug resistance.

In the tumor microenvironment, factors such as the Warburg effect, hypoxia, pH deregulation, and cell metabolism influence the availability of succinylation donors and regulators, which in turn affect the degree of protein succinylation^[15–20]^. Mitochondrial metabolism, for instance, modulates succinyl-CoA availability[19]. Succinylation is inhibited by SIRT5, SIRT7, and CoBb as desuccinylase[20]. Positive regulation of succinylation is achieved by succinyltransferases such as KAT2A, alpha-KGDHC, HAT1, and CPT1A. Changes in tumor microenvironment alter the role of these enzymes as mediators of succinylation, alter the degree of succinylation, and modify metabolism.

**HIGHLIGHTS**

- Succinylation is a newly discovered PTM: It explains the mechanism of protein succinylation, alters the structure and activity of proteins, and affects their functions.
- Regulation of succinylation by various succinylating factors: The regulation of protein succinylation levels by succinylating donors, desuccinylating enzymes, succinyltransferases, and other modified enzymes was analyzed.
- Metabolic reprogramming and regulation of immune processes: This highlights the role of succinylation in regulating key metabolic nodes such as the tricarboxylic acid cycle, oxidative phosphorylation, amino acid metabolism, and fatty acid synthesis in tumor cells, as well as its regulatory role in immune metabolism.
- Impact on tumorigenesis: It elaborates on the modification of cancer cell protein succinylation by desuccinylase and succinyltransferase in tumor cells, thereby influencing tumor progression and metastasis.
- Therapeutic Implications: The latest research on cancer treatment targeting succinylation has been summarized. Targeting succinylation pathways may provide new strategies for cancer therapy

### The base of succinylation modification

Succinylation, an evolutionarily conserved post-translational modification (PTM) found across species, is closely associated with various physiological processes[21]. Protein succinylation serves as a key regulatory mechanism for numerous cellular activities such as proliferation, growth, differentiation, metabolic processes, and potentially tumorigenesis[22]. Of them, succinylation regulates protein function and cellular processes by regulating epigenetics and gene expression through metabolism. For instance, SIRT5, through de-succinylation of CPT2, increases the metabolic efficiency of cardiomyocytes and alleviates diabetic cardiomyopathy lipotoxicity^[23,24]^. SUCLG2 deletion has been speculated to elevate the succinylation level of mitochondrial proteins, impairing the function or stability of critical metabolic enzymes, thus modifying mitochondrial function in LUAD cells[25]. Notably, succinylation of histone H3, e.g., H3K122succ, directly regulates nucleosome stability and, in turn, transcriptional fate[26]. In essence, succinylation plays a critical role in cancer and metabolic diseases by dynamically modulating proteins through metabolite-driven mechanisms.

### The core mechanism of TME regulation of succinylation

Succinylation is a unique protein post-translational modification process that involves the modification of lysine residues in target proteins by metabolically derived succinyl-CoA[27]. This modification can be driven by succinyl-CoA, succinate, or other succinyl metabolites to achieve dynamic regulation of succinylation in cells[28]. As a non-enzymatic modification mechanism, the dynamic balance of succinylation is regulated by multiple factors, such regulation is influenced by critical factors, including succinyl donor availability, intracellular pH balance, and cellular redox status[29]. In addition, the metabolic state of the cell, environmental conditions such as temperature and pH, and signal transduction pathways within the cell also play an important role in maintaining the succinylation balance^[30,31]^. Therefore, succinylation is not only one of the important post-translational modifications in cells, but also the complexity of its regulatory mechanism reflects the precise regulation process carried out by cells under different physiological and pathological conditions.

### Escape of immunity

Immune evasion is the central mechanism through which typical tumor cells and pathogens evade the immune system. It is a term to describe how cancer cells or infections have the ability to escape recognition and elimination by the immune system using many mechanisms in an attempt to survive and propagate within the host. Shared pathways consist of down-regulation of antigen presentation molecules to hide their own characteristics, overexpression of immune checkpoint molecules to inhibit T cell activity, release of immunosuppressive factors, or the attraction of regulatory T cells and MDSCs function to inhibit host immune activity and promote immune evasion[32]. Tumors are also able to reengineer the immune suppressive microenvironment through metabolic changes (such as the accumulation of lactate and depletion of tryptophan) or epigenetic changes (e.g., DNA methylation and histone succinylation)[33]. These pathways synergistically act to prevent the immune system from being able to effectively eliminate faulty cells, thus leading to disease progression. Immunotherapeutic targeting of immune escape has become a central approach to cancer treatment.

We searched PubMed with keywords including succinylation, metabolic reprogramming, and concepts related to tumor metabolism and immune regulation (e.g., “cell metabolism,” “immune,” etc.). In the literature on the regulation of succinylation, the literature published in the last 5 years was given priority to ensure the timeliness of the evidence. The underlying literature that originally proposed or defined the relevant concept was also cited to ensure the authority of the concept. Exclusion criteria were non-English literature, case reports, or studies of low scientific quality. This work complies with the TITAN Guidelines 2025 for transparent AI use in research[34]. We declare AI was used exclusively for language refinement during manuscript revision. All scientific content, data interpretation, and conclusions are human-generated and validated.

## Mechanisms by which succinylation regulates the NF-κB pathway for immune escape

The NF-κB pathway is pivotal in various pathophysiological processes such as inflammation, immune regulation, and tumor immune evasion[35]. Recent evidence indicates that lysine succinylation may play a potential role in the regulation of the NF-κB pathway activity, especially the structure and function regulation of its central members IκBα and RelA (p65)^[36,37]^. Succinylation of lysine residues on IκBα, a natural inhibitor of the NF-κB complex, induces conformational changes that facilitate its recognition by the IKK complex for phosphorylation, ubiquitination, and subsequent degradation[38]. This mechanism can significantly accelerate IκBα degradation, reducing its inhibitory duration, promoting rapid translocation of the NF-κB complex from the cytoplasm to the nucleus, and enhancing transcriptional activity. RelA (p65) is a key transcription factor found in NF-κB heterodimers, and its action is regulated through various post-translational modifications. Experiments have shown that succinylation of RelA not only enhances its nuclear localization and DNA binding capability but also seemingly suppresses its degradative or dephosphorylation process, prolonging NF-κB pathway activation time[39]. Such a modification mechanism can finally lead to the long-term expression of a set of inflammatory cytokines and immunomodulatory cytokines, offering a molecular explanation for the tumor cells to establish the immunosuppressive microenvironment.

Chemokines have a pivotal function in the mediation of cell migration of immune cells and direction of infiltration. As molecules that regulate the elements of the tumor immune microenvironment (TIME), the dysregulation of chemokines is typically intimately linked with immune evasion of tumor[40]. Recent studies have revealed that succinylation, as a major lysine post-translational modification, not only has an essential role in the regulation of metabolism, but it also participates in the regulation of transcription factor activity and chromatin state, enhances expression of a variety of immunosuppressive chemokines, and leads to the accumulation of immunosuppressive cells in the tumor microenvironment. The immune microenvironment is immune-favorable for tumor immune escape[41]. Among these chemokines, CCL2 and CXCL1 are prototypic immunosuppressive chemokines which have been well explored. CCL2 may effectively support MDSCs and TAMs recruitment and induce tumor immunosuppression. At the level of molecular mechanism, NF-κB is considered the master transcriptional regulator of the CCL2 promoter[42]. Succinylation and inhibition of KEAP1 by fumarate suppressing subsequent ubiquitin proteasomal degradation of CHD6. Fumarate-induced succinylation has been shown to activate KEAP1 inactivation, inhibiting ubiquitin-proteasome-mediated degradation of CHD6 protein. The stable expression of CHD6 protein can engage in constituting a transcription complex with NF-κB subunit p65, promoting the assembly of proinflammatory enhancers and NF-κB-mediated CCL2 transcriptional activation[43]. Additionally, this process also interacts with other NF-κB signaling molecules activated by succinylation to work together to facilitate the sustained expression of immunosuppressive chemokines such as CCL2 and promote the establishment of an immunosuppressive microenvironment.

## Succinyl-CoA levels regulate succinylation

Succinyl-CoA is a significant metabolic intermediate and is involved in a number of biochemical reactions such as the TCA cycle, biosynthesis of porphyrins, and catabolism of odd-chain fatty acids and certain branched-chain amino acids. Succinyl-CoA is produced by TCA cycle or amino acid metabolism[44] and can either cross the mitochondrial membrane or be synthesized outside the mitochondria[30]. Succinyl-CoA brings about the succinylation of the lysine residues in pH- and concentration-dependent manner[45] forms a complex with the NH3-1 group at the lysine side chain of the protein, and donates the succinyl group (–CO–CH2–CH2–CO2H) to the lysine residue to change the structure and function of the protein, resulting in the succinylation of the protein[2].

### Formation and accumulation of succinyl-CoA

Succinyl-CoA, as the main acyl donor of succinylation, plays a central role in this modification process[25]. Succinyl-CoA is a key intermediate in a variety of metabolic pathways. It is widely involved in the TCA cycle, the biosynthesis of porphyrins, and the catabolism of odd-chain fatty acids and branched-chain amino acids. Succinyl-CoA is mainly generated by the TCA cycle or amino acid metabolism in mitochondria[30], and its dynamic changes in intracellular concentration are closely related to the level of protein succinylation. In the TCA cycle and the metabolic process of the respiratory chain, the synthesis and transformation of succinyl-CoA depend on the synergistic action of a variety of mitochondrial enzymes[44], including Alpha-ketoglutarate dehydrogenase complex (alpha-KGDH), succinyltransferase (SCS), and succinate dehydrogenase (SDH)[46]. When TCA cycle function is impaired, KGDH and SDH activities are usually decreased, while SCS activity is significantly increased[46]. The remodeling of this metabolic enzyme activity not only inhibits the production of succinyl-CoA but also promotes its rapid conversion to succinate, which leads to a decrease in the overall level of succinyl-CoA in mitochondria and ultimately inhibits the succinylation modification of proteins.

Succinyl-CoA biosynthesis depends mostly on Alpha-ketoglutarate dehydrogenase complex (KGDHC) in the TCA cycle, E1k (Alpha-ketoglutarate dehydrogenase), E2k (dihydrosulfyl succinyl transferase), and E3 (dihydrosulfyl dehydrogenase)[44]. Amino acid metabolism provides succinyl-CoA precursors for heme biosynthesis, and KGDHC catalyzes its biosynthesis[29]. Thus, KGDHC directly affects the level of protein succinylation through the metabolism regulation of succinyl-CoA or directly participates in succinylation by substrate supply regulation. Succinylation is a covalent modification reaction between succinyl-CoA and -amino group (–NH3+) of protein lysine residues[2], leading to protein conformational and functional changes. The modification process is shown to be substrate concentration and pH environment dependent, reflecting its metabolic state-regulated nature[45].

In tumor cells, there is the overall phenomenon of “aerobic glycolysis,” namely the Warburg effect[47]. Cells maintain their major source of energy as glycolysis despite the presence of a sufficient oxygen supply. Whereas, conversely, augmented mitochondrial metabolic rate and heightened TCA cycle flux lead to the buildup of different metabolic intermediates comprising enhanced expression or activity of crucial enzymes such as KGDHC, SCS, SDH, and succinyl-CoA synthetase, which finally lead to succinyl-CoA accumulation[48]. This increase in succinyl-CoA levels can elevate the succinylation protein modification, and then be involved in the regulation of metabolic reprogramming and functional status of cancer cells.

The succinylation modification has also been reported to take place in Saccharomyces cerevisiae. Alpha-ketoglutarate mitochondria is catalyzed by the Alpha-ketoglutarate dehydrogenase complex (Kgd1, Kgd2, and Lpd1) to succinyl-CoA, which is then catalyzed by succinyl-CoA synthetase composed of Lsc1 and Lsc2[49]. Deletion of Kgd1 significantly reduces protein succinylation in yeast cells, while deletion of Lsc1 enhances succinylation, suggesting thatsuccinyl-CoA accumulation plays a crucial role in succinylation regulation^[30,49]^. Additionally, mammalian cell SUCLG2 (β subunit of GDP-type succinyl-CoA synthetase) has the ability to catalyze succinyl-CoA hydrolysis and the succinylation modification process in mitochondria, once again pointing to the central regulatory role of succinyl-CoA metabolic dynamics in the regulation of protein succinylation[25].

Besides regulating the succinyl-CoA metabolic enzyme network directly, dynamic balance in the mitochondrial succinate metabolic network also significantly impacts protein succinylation modification. Furthermore, SDH is also a crucial metabolic node connecting the tricarboxylic acid cycle and oxidative phosphorylation, and its catalytic activity modulates the steady-state level of the succinate/fumarate metabolic pathway directly. Maintenance of such homeostasis is not merely linked to the efficacy of energy metabolism but has the potential to drive the overall direction of protein post-translational modification by regulating the balance of succinyl-CoA generation and consumption.

### Succinate dehydrogenase inhibition

Succinate dehydrogenase (SDH), a critical part of the mitochondrial respiratory chain and the TCA cycle, is a heterotetrameric group of enzymes derived from the subunits SDHA, SDHB, SDHC, and SDHD encoded by autosomal genes SDHA (5p15.33), SDHB (1p36.13), SDHC (1q23.3), and SDHD (1q23.3)[50]. These subunits are located in the inner mitochondrial membrane and form mitochondrial complex II. SDH is necessary for cell bioenergetics, bridges two pathways TCA cycle and oxidative phosphorylation (OXPHOS)[50]. SDH facilitates succinate conversion to fumarate. Hence, SDH contributes energy production via electron transport chain^[50,51]^. The oxidation process of SDH relies on the cofactors FAD and NAD+[52].

In normal cellular metabolism, SDH is largely in its reduced state. In cellular hypoxia, SDH can be inhibited to enable the build-up of succinate. Lipopolysaccharide (LPS) can also decrease NAD+, which leads to FAD reduction and inhibition of SDH activity. Reduction in the levels of NAD+ disturbs the function of deacetylase SIRT3, which confers deacetylase activity to SDHA, catalytic subunit of SDH, and thus contributes to its activation as well as regulation of intracellular succinate levels[53]. Lack of SDH function prevents succinate to fumarate, leading to high succinate and succinyl-CoA levels that lead to excessive succinylation^[19,54]^. Protein succinylation, such as glyceraldehyde-3-phosphate dehydrogenase or malate dehydrogenase, can be involved, and enzymes during succinate buildup can lead to increased succinylation^[2,55,56]^. Moreover, the build-up succinate is converted to succinyl-CoA by succinyl-CoA synthetase[56], promoting additional protein succinylation[19].

SDH inhibition causes succinylation and succinyl-CoA to build up, thus succinylation of lysine is enhanced. This, under SDH deficiency, may cause a disruption of genomic location of succinylation chromatin modification, especially at the promoter region of genes[54]. Promoter region succinylation has been found to be most prominent in chromatin by SDH knockdown[54]. Genome-wide association study shows that such promoter-targeting succinylation modification differs significantly with changes in the transcriptome. At the same time, epigenetic marks H3K4me3 and H3K27me3 are also perturb in the same region. This suggests that TCA cycle metabolites can regulate gene expression through synergistic epigenetic regulation mode[54]. Therefore, when the function of TCA cycle is impaired, especially when SDH deficient, the addition of succinate and succinyl-CoA increases to increase the frequency of succinylation modification, which also regulates gene expression synergistically by succinylation modification gene promoter regions^[19,54]^.

On the basis of the dynamic balance of the succinyl-CoA metabolic pathway regulating protein succinylation modification, the feedback regulation of dessuccinylase activity further constitutes a bidirectional regulatory network. Studies have shown that mitochondrial NAD+-dependent dessuccinylase SIRT5 can form a closed-loop regulation of metabolism and modification with succinyl-CoA synthetase (such as SUCLG2) by catalyzing the hydrolysis reaction of lysine succinyl group[57] and a closed-loop regulation of metabolism-modification with succinyl-CoA synthetase such as SUCLG2[25]. When succinyl-CoA levels accumulate due to abnormal metabolic enzyme activity (such as SDH inhibition or Lsc1 deletion), SIRT5 expression or activity may be adaptively regulated through metabolite sensing mechanisms (such as changes in NAD+/NADH ratio)[58], thereby synergistically maintaining the homeostasis of intracellular succinylation modification.

### Regulation of the dessuccinylase SIRT5

Sirtuin 5 (SIRT5) is a well-established desuccinylase primarily found in the mitochondrial matrix and whose desuccinylation is dependent on NAD+. SIRT5 recognizes and binds succinylated substrates, using NAD+ as a covalent cofactor to catalyze succinyl group removal. This process allows the substrate to revert to its original unmodified state or triggers a change in its chemical status[57].

#### Structural characteristics and catalytic mechanism

The substrate binding site of SIRT5 contains three hydrophobic residues (Leu227, Phe223, Val254) for the acyl-lysine binding pocket and two nonhydrophobic residues (Arg105, Tyr102) for the acyl-lysine moiety with net negative charge[57]. Ala86 is important in the formation of even larger lysine acyl binding pocket, which allows SIRT5 to select for short chains carboxyl groups (e.g., succinyl and malondialyl)[18].

SIRT5 catalytic mechanism requires NAD+[59], possessing a Zn^2+^ binding domain and a Rossmann folding domain, which are together the substrate binding site and NAD+ binding site[60]. The recognition of succinylated lysine residues by sequence or structure is achieved by SIRT5 in dessuccinylation[61]. Bound to the substrate, NAD+ is a covalent catalyst in the role of an electron donor and to form an intermediate (ADP-ribose) at the enzyme active site. The conserved cysteine residue of the active site of SIRT5 becomes covalently bonded to the succinyl group of the succinyllysine residue, followed by NAD+ conversion to nicotinamide and release from the complex. Later. Through the action of water molecules, the succinyl moiety is eliminated from the cysteine residue in the active site of the enzyme, thus releasing free succinate and the initial unmodified state of the substrate[62].

#### Regulation of NAD+ and SIRT5 activity

SIRT5 activity is significantly dependent on the intracellular level of NAD+[59]. NAD+ not only functions as an SIRT5 cofactor to catalyze the process of dessuccinylation but also acts in the catalysis process to generate nicotinamide and ADP-ribose. High NAD+ concentrations increase SIRT5 enzyme activity through adequate supply of cofactors[62], whereas a lower concentration of NAD+ reduces the efficiency of SIRT5 dessuccinylation through cofactor deficiency[63]. Cellular energy state, or rather the cellular metabolic state, may have a direct effect on the amount of NAD+, and thus indirectly on the activity of SIRT5[58]. For example, hypoxia is a ubiquitous feature of the tumor microenvironment[64]. NAD+ serves as a vital link between mitochondrial energy metabolism and cellular signal transduction pathways. Under hypoxic conditions, HIF1α enhances the transcription of LDHA, a pyruvate catalytic enzyme to lactate, thereby enhancing the cytoplasmic level of NAD+[64], which can enhance SIRT5 activity. Under hypoxic conditions, HIF-1α enhances transcription of LDHA and elevates the concentration of NAD+. The ratio of NAD+/NADH is an important contributor to SIRT5 activity. Hypoxia-induced inhibition of the TCA cycle has a significant impact on this ratio and more NAD+ for availability as a cofactor, favoring SIRT5 activity, whereas a reduced ratio suppresses SIRT5 activity^[65,66]^, impacting lysine succinylation.

#### Tumor microenvironment and regulation of SIRT5

The metabolic state of the TME also has an extensive impact on SIRT5 activity. Tumor cells rely on reprogramming of aerobic glycolysis, mitochondrial respiration, glutamate metabolism, and lipid metabolism^[67,68]^, that is closely interlinked with the Warburg effect. Glutamine content in TME is of critical significance, with its role not only in Alpha-ketoglutarate (α-KG) production but also in maintaining the homeostasis of mitochondrial membrane potential as well as reduced glutathione[69]. Under metabolic stress caused by glutamine depletion, SIRT5 interacts in stable complex with malic enzyme 2 (ME2) through its mitochondrial targeting sequence, modulating the activity of ME2 by dessuccinylation. During the process, SIRT5 deambers at the K346 site on ME2 in an NAD+-dependent manner[70]. Therefore, the levels of metabolites NAD+ and glutamine in TME modulate the activity of SIRT5 and hence modulate succinylation.

#### Synergistic effect of NAD+ and glutamine concentrations

The activity of SIRT5 in the TME is collectively regulated by the levels of NAD+ and glutamine^[67,68]^. High glutamine concentrations can help in desuccinylation by increasing binding of substrates to SIRT5, and low glutamine concentrations may hinder the process. In fact, SIRT5 activity in TME is regulated not only by NAD+ concentration, but also by reprogramming glucose/glutamases metabolism^[70,71]^.

SIRT5 is a desuccinylase that regulates cell metabolic regulation, and is dependent on the NAD+ concentration. NAD+ and glutamine are important for tumor cells and regulate the metabolism of tumor cells by modulating the activity of SIRT5[70]. SIRT5 regulatory pathways may provide new insights into metabolic reprogramming of cancer cells and may provide targets for future anticancer treatment.

### Effects on the metabolic state of the cell

As a significant post-translational modification, the dynamic balance of succinylation is intricately regulated by numerous factors[29]. In addition to the participation of metabolites like NAD+ and glutamine, cellular metabolic states, environmental conditions, as well as intracellular signaling mechanisms, also significantly regulate the amount of succinylation^[30,31]^.

#### Effect of cellular metabolic state on succinylation

The extent of protein succinylation is directly governed by cellular metabolic status and varies significantly with metabolic fluctuations. For example, upon inhibition of glycolysis (e.g., with 2-deoxyglucose) or glutathione depletion (e.g., with iodoacetic acid), TCA cycle activity is reduced along with the degree of succinylation. In addition, electron transport chain blockage (e.g., antimycin effect), defective ATP synthetase (e.g., treatment with oligomycin), and low oxidative phosphorylation of uncouplers (e.g., carbonyl cyanide, m-chlorophenyl hydrazine, or tyrphostin) inhibit the succinylation process[31]. The metabolic status of the cell directly affects the degree of succinylation. The degree of succinylation is quite different when cells are in different states of metabolism. For example, when glycolysis is inhibited (e.g., when 2-deoxyglucose is used) or glutathione is reduced (e.g., with the use of iodoacetic acid), activity of TCA cycle falls, and the level of succinylation also declines. In addition, inhibition of electron transport chain (e.g., action of antimycin), defective operation of ATP synthetase (e.g., use of oligomycin), and impaired oxidative phosphorylation by uncoupling agents (e.g., carbonyl cyanide, m-chlorophenyl hydrazide, or tyrphostin) also inhibit the process of succinylation[65].

#### Effect of metabolites on succinylation

A decrease in succinate dehydrogenase leads to the accumulation of succinate and succinyl-CoA, which promotes histone succinylation. Studies have shown that proteins involved in glycolysis, TCA cycle, fatty acid metabolism, ketone metabolism, heat shock response, solute transport, ATP synthesis, amino acid synthesis, and electron transport chain are all significantly affected in succinylated cells. The dynamic changes of succinylation in different metabolic states indicate its important role in the metabolic reprogramming of cells^[28,65]^.

#### Positive regulation by succinyltransferase and negative regulation by dessuccinylase

Succinylation is also regulated by a broad family of enzymes^[48,72,73]^, including the activity of succinyltransferases and dessuccinylases. Lysine acetyltransferase 2A (KAT2A) also catalyzes succinyl group transfer, thereby regulating H3K79 succinylation and glycolysis[74]. In addition, KAT2A has the ability to up-regulate β-catenin stability. Histone acetyltransferase 1 (HAT1) is another succinyl transferase that performs lysine succinylation modifications on histones and non-histones and also plays a role in epigenetic regulation and gene expression at H3K122[75]. Enzymes such as alpha-KGDHC and CPT1A also contribute to the regulation of protein succinylation[72]. They also play a role in intracellular metabolic activity through increased levels of succinylation. Dessuccinylase is also an important regulator in negatively modulating the level of succinylation. SIRT5 and SIRT7, as desuccinylase[27], can decrease the level of succinylation by removing the amber group from the protein. CobB also has been shown to exhibit the same activity of desuccinylation; thus, these desuccinylase enzymes regulate the balance of succinylation[29].

**Figure 1.**
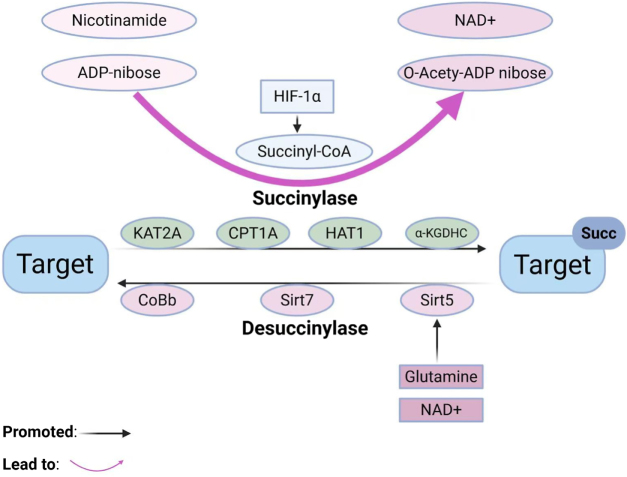
Lysine succinylation is catalyzed by succinyltransferases KAT2A, CPT1A, HAT1, and alpha-KGDHC, which covalently modify the lysine residues of target proteins with succinyl groups from succinyl-CoA. This process is regulated by hypoxia-inducible factor 1α, promoting the generation of NAD+-bound O-acetyl-ADP ribose, thereby regulating cellular metabolism and signal transduction. Desuccinylation is mediated by desuccinylases CoBb, SIRT7, and NAD+ and glutamine-dependent SIRT5, dynamically regulating the function of target proteins through hydrolysis of the modification. Succinyl-CoA, Succinyl coenzyme A HIF-1α, Hypoxia-inducible factor 1 alpha alpha-KGDHC, Alpha-ketoglutarate dehydrogenase complex CoBb, Desuccinylase enzyme SIRT, Sirtuin family proteins. This figure was created by Biorender.Com.

The cellular metabolic status, extracellular milieu, and many intracellular enzymes regulate the dynamic equilibrium of succinylation^[48,72,73]^. The fluctuation in metabolite concentration such as succinic acid and succinyl-CoA variation, alteration in oxygen levels, and coordination of metabolism processes such as glycolysis and TCA cycle, all have a significant impact on the degree of succinylation. In addition, the action of succinyltransferases (such as KAT2A, HAT1, etc.) and dessuccinylated enzymes (such as SIRT5, SIRT7, CoBb, etc.) together guarantees fine regulation of succinylation modification^[48,73,75]^. Insight into such processes provides important information regarding cellular metabolism and related pathologic processes, such as tumorigenesis and progression (Fig. 1) (Table 1).

**Table 1:** Functions, effects and influencing factors of regulatory factors affecting succinylation

Regulatory factors of succinylation	Function	Effect of action	Influencing factors	References
Succinyl-CoA	Succinyl-CoA, as a substrate for succinylation, reacts with the NH3-1 group on the lysine side chain of proteins in a pH- and concentration-dependent manner, transferring the succinyl group to the lysine residue to change the structure and function of proteins.	Succinylation can be promoted when excess succinyl-CoA accumulates	TCA, respiratory chain metabolism and intracellular metabolites	^[25,27,76]^
SDH	SDH catalyzes the conversion of succinate to fumarate and participates in the energy generation process through the electron transport chain.	When SDH is inhibited, it leads to abnormal accumulation of succinic acid and then promotes the accumulation of succinyl-CoA to accelerate succinylation	Hypoxia, NAD+, LPS	^[53,77]^
Sirt5	Dessuccinylase, is mainly localized in the mitochondrial matrix. SIRT5 and Sirt7 recognize and bind to the succinylated protein substrate, catalyzing the removal of the amber group from the substrate, restoring the unmodified state of the substrate or altering its chemical environment.	An increase in the concentration of dessuccinylase inhibited protein succinylation	Aerobic glycolysis, mitochondrial respiration, glutamate metabolism, and metabolic reprogramming of lipids.	[27]
Sirt7				
KAT2A	It catalyzes the transfer of succinyl groups, thereby regulating the succinylation of H3K79 and promoting glycolysis.	It can bind succinyl-CoA to transfer succinyl group to histone H3 and promote glioma proliferation.	Hypoxia, inflammatory factors, stromal cell and ECM signaling	^[78,79]^
HAT1	As a succinyltransferase, it catalyzes succinylation of histone and non-histone proteins and is critical for the regulation of epigenetics and gene expression at H3K122.	By promoting the succinylation modification of histone, it enhances its enzyme activity, and accelerates glycolysis and cancer cell proliferation.	Hypoxia, acetyl-CoA level, and proinflammatory cytokines such as TNF-α	^[72,75]^
CPT1A	It can play the role of succinyltransferase in vivo and in vitro, and regulate substrate proteins and related metabolic processes.	Cpt1a-mediated succinylation modification of enolase 1 can inhibit its activity and promote cell proliferation in glutamine-depleted state.	Fatty acid metabolic reprogramming, hypoxia, and HIF-1α regulation	^[80,81]^
α-KGDHC	α-KGDHC is an α-ketoglutarate dependent succinoacyltransferase that mainly functions in mitochondria.	Inhibition of α-KGDHC reduced both the levels of mitochondrial matrix and mitochondrial protein succinylation.	Warburg effect, abnormal accumulation of metabolites in the TCA cycle, hypoxia leads to an inhibitory effect of HIF-1α	^[28,29]^

## Metabolic reprogramming mediated by succinylation

Metabolic reprogramming is one of the most important biological features of tumorigenesis, as it is one of the main biological characteristics of tumorigenesis that distinguishes cancer cells from normal cells[82]. Cancer cells can regulate and remodel metabolic pathways in order to obtain the high energy biosynthesis precursors needed for their malignant biological characteristics, such as continuous proliferation, invasion, and metastasis. Not only does metabolic reprogramming provide sufficient energy to cancer cells, but it also produces a cascade of critical metabolic intermediates involved in the biosynthesis of macromolecules such as nucleic acids, proteins, and lipids to sustain the rapid growth of tumor cells. In addition, some of the metabolic alterations will also interfere with the apoptosis signaling pathways in a variety of mechanisms that enable cancer cells to become resistant to programmed death and increase the frequency and development of tumors[83].

Metabolic reprogramming is characterized by several hallmark features: faster glycolysis with reduced mitochondrial respiration, increased fatty acids biosynthesis, increased glutamine addiction and altered amino acids utilization[84]. Together, these changes construct a cancer cell-specific metabolic network.

Succinylation is a new PTM[27]. Compared to methylation (14 Da) and acetylation (40 Da), succinylation produces a larger mass change (100 Da) and could convert the positively charged side chain of lysine to a negatively charged moiety. As a result, the charge state of the protein experiences a far-reaching shift (usually from +1 to −1), greatly altering the protein’s conformation, function, and metabolism[2].

A typical source of succinylation is the non-enzymatic modification of fumarate, which can covalently bind to the thiol group of the cysteine residue of a protein to generate S-2-succinyl-diacid (2SC), a process that is thought to be more active under stress or pathological conditions[85].

Succinylation is crucial for the metabolic reprogramming of cells, especially in tumor-related metabolic pathways with high functional relevance. Since its precursor molecule succinyl-CoA is mainly produced in mitochondria, succinylation mainly regulates key metabolic pathways in mitochondria, including but not limited to tricarboxylic acid (TCA) cycle, electron transport chain, fatty acid β-oxidation, and ATP synthesis[86]. In addition, succinylation is also widely involved in a variety of metabolic processes such as glucose metabolism, amino acid degradation, ketogenesis, and urea cycle^[87,88]^, thereby regulating cellular energy status, REDOX balance, and metabolic adaptability, and promoting or maintaining the metabolic advantages of tumor cells.

Succinylation has been demonstrated to modulate the enzymatic activity and stability of numerous metabolic enzymes, thereby redirecting metabolic flux and pathway regulation[29], which is essential for guaranteeing tumor cell metabolic plasticity^[89,90]^. Succinylation has therefore been targeted as one of the essential metabolic reprogramming regulation mechanisms.

Metabolic reprogramming is the signature of cancer cells. In parallel, succinylation has been revealed to occur as a key regulator in modulating mitochondrial energy metabolism, especially in Krebs cycle and OXPHOS pathway. Succinylation alters the structure and charge pattern of protein and thus affects its stability and catalytic activity, and therefore remolds the metabolic network in cancer cells. As a critical mitochondrial dessuccinylase, SIRT5 regulates the succinylation status of several important metabolic enzymes and is vital to cellular metabolic homeostasis.

### Succinylation affects TCA

The TCA cycle is one of the core energy generation pathways in mitochondria, which provides cells with NADH, FADH_2_, and other reducing equivalents to drive the electron transport chain[91], and simultaneously generates a variety of biosynthetic precursors. Studies have shown that a variety of TCA cycle key enzymes can undergo lysine succinylation modification[30]. These enzymes include citrate synthase (CS), aconitate hydratase (ACO), pyruvate dehydrogenase complex (PDH), succinate dehydrogenase (SDH), Alpha-ketoglutarate dehydrogenase complex (KGDHC), isocitrate dehydrogenase (IDH), fumarate dehydrogenase, and malate dehydrogenase.

PDH is the regulator of glucose oxidation by catalyzing the conversion of pyruvate, the main end product of glycolysis, into acetyl-CoA and CO2 by the pyruvate dehydrogenase complex. PDH catalyzes substrates for the TCA cycle and is one of the lysine succinylation targets[92]. There are three chemical replicates of every one of the enzymatic subunits. There are E1 (branched-chain α-keto acid decarboxylase), E2 (lipoamide acyltransferase), and E3 (lipoamide dehydrogenase). Of these, E2 is succinylated at Lysine 278[93], suppressing PDH activity and energy production. Recent studies suggest that lysine succinylation can increase PDH activity in an E1-phosphorylation independent manner[94]. In this manner, succinylation controls the shift from glucose oxidation to glycogen rebuilding by regulating PDH activity, which in turn affects the gluconeogenic cycle.

Succinylation of Lys100, Lys199, and Lys242 in isocitrate dehydrogenase (IDH) can interfere with substrate binding through conformational changes, inhibit catalytic activity, and reduce TCA cycle efficiency^[2,92]^. Lys242, in particular, when succinylated, creates a novel salt bridge with Glu238 and Asp279, which suggests it is a critical participant in the regulation of IDH function.

**Figure 2.**
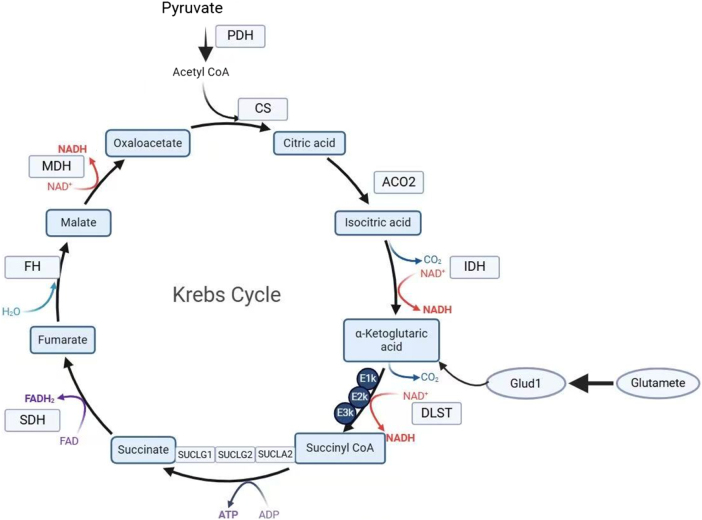
Succinylation of the tricarboxylic acid cycle under normal conditions leads to a significant drop in activity for most of the important enzymes. Succinylation of CS inhibits its catalytic condensation of oxaloacetate and acetyl-CoA, which limits access to the cycle. Succinylation of IDH inhibits its catalytic decarboxylation of isocitrate to Alpha-ketoglutarate and NADPH. Succinylation of DLST in the alpha-KGDH complex inhibits decarboxylation of Alpha-ketoglutarate to succinyl-CoA. Succinylation of SDHA inhibits oxidation of succinate to fumaric acid. FH and MDH succinylation suppressed fumarate hydration and malate dehydrogenation, respectively. For a few enzymes, such as GLUD1, succinylation may enhance their activity, which allows for oxidative deamination of glutamate to Alpha-ketoglutarate, providing an alternative substrate for TCA. For multi-subunit complexes such as PDH, alpha-KGDH, SDH, succinylation may disrupt inter-subunit interactions and complex assembly or stability. CS, Citrate Synthase IDH, Isocitrate Dehydrogenase alpha-KGDH, Alpha-Ketoglutarate Dehydrogenase DLST, Dihydrolipoamide Succinyltransferase SDHA, Succinate Dehydrogenase Complex Subunit A FH, Fumarase MDH, Malate Dehydrogenase GLUD1, Glutamate Dehydrogenase 1 PDH, Pyruvate Dehydrogenase SDH, Succinate Dehydrogenase. This figure was created by Biorender.Com.

Succinylation of Cys178 in the DLST subunit of the Alpha-ketoglutarate dehydrogenase complex (KGDHC) disrupts intermolecular hydrogen bonds, causing a conformation shift and a catastrophic inhibition of the enzymic activity of the enzyme complex, and thus inhibits the formation of succinyl-CoA via the TCA cycle. For the same reason, this modification also inhibits the activity of succinyl-CoA ligase (SUCLA) in substrat-level phosphorylation, and thus worsens mitochondrial energy deficiency. KGDHC is a TCA cycle rate-controlling step, and succinylation of one subunit of KGDHC in TCA, dihydrolipoyllysine-residue succinyltransferase (DLST), has a significant impact on the metabolic functions of mitochondria[95]. Studies have shown that mature DLSTS in both mice and humans contain two cysteines, Cys178 and Cys37. After the amylation of Cys178 by molecular dynamics simulations, the interaction pattern of some regions of the protein was altered. The stable hydrogen bond formed between Arg358 and asp356 is broken, so that DLST succinylation reduces KGDHC activity. KGDHC-derived succinyl-CoA production defects thus reduce the transformation of succinyl-CoA to succinate within the tricarboxylic acid cycle. Succinyl-CoA ligase (SUCLA) catalyzes the conversion of succinyl-CoA to succinate during the TCA cycle, and this enzyme is responsible for the synthesis of GTP or ATP by substratum-level phosphorylation (SLP)[96]. Therefore, specific succinylation of dihydroacyl-residue succinylase transferase (DLST) irreversibly reduces the activity of Alpha-ketoglutarate dehydrogenase complex (KGDHC). This persistent defect worsens mitochondrial OXPHOS-derived ATP defects by limiting compensatory substrate level phosphorylation (SLP) of succinyl-CoA ligase[97] (Fig. 2).

### Succinylation affects oxidative phosphorylation

The electron transport chain (ETC) and oxidative phosphorylation are critical to the upkeep of cellular ATP supply. Studies have revealed that certain subunits of ETC complexes I to V can succinylate (changing protein-protein interactions between subunits), which affects mitochondrial respiratory chain efficiency and rate of ATP synthesis[98].

SDH is involved in mitochondrial OXPHOS[99]. SDHA subunit of SDH, a part of mitochondrial complex II in the electron transport chain, is identified as a target for succinylation controlled by SIRT5[94]. Succinylation at Lys 250 regulates SDH activity and releases proinflammatory cytokines, which influence cell metabolism. SDHA succinylation can positively affect SDH activity, and over-activates SDH after higher succinylation. This hyperactivation results in a significant elevation in mitochondrial reactive oxygen species (mtROS) levels, disrupting cellular homeostasis and promoting oxidative stress. Succinylation of SDHA can activate proinflammatory cytokine release and increase mtROS by hyper-activating SDH, which affects cell metabolic status.

Serine-lactamase-like protein (LACTB) is the predominant substrate of OXCT1-mediated succinylation. LACTB is fibrous structure of mitochondria intermembrane space and plays important roles in mitochondrial structure and function, control by mitochondrial PISD protein and metabolite PE. Catastrophic succinylation of LACTB at K284 abolishes its proteolytic activity, increases mitochondrial membrane potential and respiratory capacity[100]. LACTB possesses proteolytic activity, and S164 is necessary for its catalytic activity. The central domain (E224-Q289) of LACTB is also flexible and functions crucially in its catalytic activity. This region can, under certain structural conditions, close the access of substrate to LACTB[101]. K284 of middle region and close to S164 is succinylated. This change changes the charge on adjacent amino acids and alterations flexibility of this region. The alteration reduces LACTB recognition of its substrate and inhibits its proteolytic activity and LACTB function. Loss of LACTB activity results in aberrant accumulation of PISD protein in mitochondria with high PE levels. OXCT1-mediated succinylation of LACTB at K284 reduces its protease activity, leading to increased PISD protein, mitochondrial membrane potential, respiration[100].

The mitochondrial inner membrane (IMM) houses a diverse array of proteins, including ion channels, solute carriers, the four respiratory chain complexes (ETC complexes I–IV), and ATP synthase. The IMM also hosts numerous peripheral proteins that bind through non-covalent electrostatic interactions rather than integrating into the membrane via transmembrane segments. These include carnitine palmitoyltransferase 2 (CPT2), mitochondrial trifunctional protein (MTP), and very long chain acyl-CoA dehydrogenase (VLCAD), which are involved jointly in -oxidation of long chain fatty acids. All three cell types are localized by cardiolipin binding in ionic conditions^[102,103]^. Succinylation of three lysine residues in VLCAD’s zinc-binding domain reduces the charge of the latter from positive to negative. This leads to inhibition of electrostatic interaction of VLCAD’s amphipathic helix with cardiolipin and consequent mitochondrial oxidative phosphorylation[102]. Succinylation thereby affects mitochondrial inner membrane structure and oxidative phosphorylation by inhibiting the character of protein charge within mitochondria.

In brief, ssuccinylation disrupts TCA cycle and OXPHOS through various targets and mechanisms and regulates cell metabolism homeostasis. SIRT5, an essential desuccinylase, is the pivotal enzyme to ensure mitochondrial metabolic integrity, inhibit oxidative stress, and restore energy equilibrium. This underscores its potential as a promising therapeutic target in the context of cancer-associated metabolic reprogramming.

### Succinylation affects fatty acid oxidation

Succinylation regulates key enzymes involved in lipid metabolism[104], such as hydroxy-coenzyme A dehydrogenase (HADH), a crucial enzyme in lipid metabolic pathways, with major responsibility for 3-hydroxybutylcoa oxidation. The findings from the study identified succinylation of HADH amino acid residue K81 at the coenzyme binding site K80[94]. Type II arginine methyltransferase PRMT5 regulates various metabolic compounds including phospholipids, fatty acids, and steroids. Succinylation modification of PRMT5 decreases its methyltransferase activity, leading to downregulation of lipid metabolism regulators such as sterol regulatory element binding protein 1a (SREBP1a), FASSN, acetyl-CoA carboxylase alpha (ACACA), Peroxisome proliferator-activated receptor gamma (PPAR), and Stearoyl-CoA desaturase (SCD)[105]. Causes abnormal deposition of fatty acids. Causes abnormal fatty acid deposition. Mitochondrial β-oxidation converts fatty acids into acetyl-CoA via a four-step enzymatic cascade: dehydrogenation (catalyzed by acyl-CoA dehydrogenases), hydration, oxidation, and thiolytic cleavage. Excessive succinylation has been shown to cause abnormal buildup of β-oxidation intermediates, impacting fatty acid metabolism through defects in β-oxidation[106]. Ketone bodies are composed of acetoacetate, β-hydroxybutyrate, and acetone[107] and are produced by the liver through fatty acid catabolism in response to glucose deficiency^[105,108]^. Ketogenesis regulates fatty acid catabolism and prevents the excessive accumulation of acetyl-CoA. Mitochondrial 3-hydroxy-3-methylglutaryl-CoA synthetase 2 (HMGCS2) is a critical enzyme in ketogenic biosynthesis and is modulated by succinylation[109]. Among the 15 succinylated lysine residues identified on HMGCS2, K83, K310, K350, K354, and K358 are highly succinylated[106]to inhibit HMGCS2 activity, down-regulate ketogenesis, and thus inhibit fat metabolism. Therefore, succinylation inhibits fatty acid oxidation through its effects on HADH, PRMT5, HMGCS2, and β-oxidation.

### Effects of succinylation on inflammation

Tumor-induced inflammation can be divided into endogenous and exogenous inflammation. Tumor-secreted inflammation occurs due to the secretion of various inflammatory signal molecules by macrophages, such as interleukin-6 (IL-6), interleukin-1 (IL-1), and tumor necrosis factor-α (TNF-α)[110]. Protein succinylation by adding succinyl groups to lysine residues is emerging as an efficient modulator of immunity and inflammation[92].

COX-2, which is a prostaglandin-synthesizing enzyme, is implicated in immunosuppressive cell recruitment and creation of an immunosuppressive tumor microenvironment. It is a key inflammatory mediator in tumorigenesis^[111,112]^. Inflammatory cytokines such as TNF-α, IL-1β, and IL-6, and other inducers such as lipopolysaccharide (LPS) can quickly stimulate COX-2 expression via nuclear factor κB (NF-κB) signaling[113]. Non-canonical prostaglandin and succinate accumulation in the tumor microenvironment, in combination with COX-2 overexpression, could enable tumor-inflammation. P23, an evolutionarily conserved protein of high evolutionary conservation encoded by the PTGES3 gene for prostaglandin E synthetase 3, is a molecular chaperone involved in the production of COX-2 by its function as a transcription factor for oncogenes of tumors. P23 is implicated in various inflammation-related signaling pathways like IL-17, TNF, chemokines, and NF-κB. The nuclear translocation of p23 is required for transcription factor activation, and succinylation plays a fundamental role in this process. Specifically, succinylation at K7/K33 causes p23 nuclear translocation to further enhance COX-2 expression[110]. Succinate, as a tumor metabolite, enhances the activation of transcription factor p23 via succinylation-induced nuclear translocation to express COX-2 in tumorigenesis to establish a well-established tumor inflammatory pathway.

**Figure 3.**
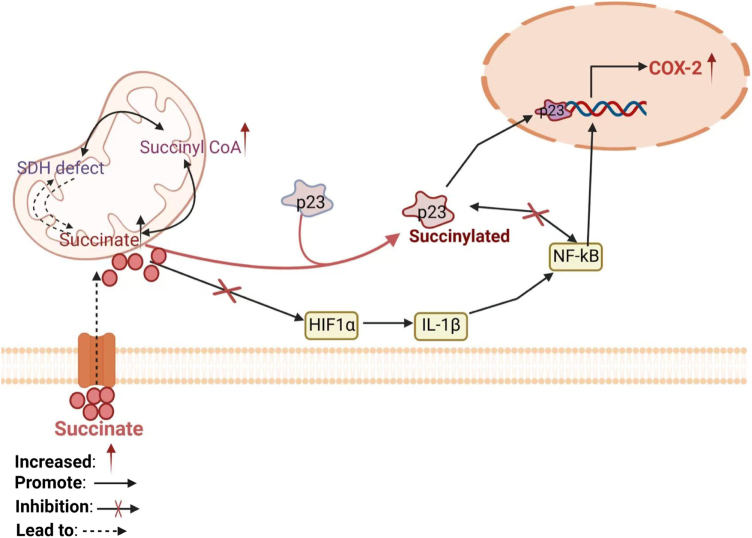
Succinate accumulates in immune cells and stabilizes HIF-1a and its receptor signaling through succinyl-CoA substrate succinylation in mitochondria, leading to the increase of IL-6, IL-1β, and NF-κB. Succinylation at K7/K33 can drive nuclear translocation of p23 to express the COX-2 gene and promote COX-2 expression. In addition, increased NF-κB could also induce additional COX-2 gene expression. Thus, this figure demonstrates that nuclear translocation of p23 driven by succinylation leads to high COX-2 expression and promotes inflammation independent of the traditional inflammatory pathway. HIF-1α, Hypoxia-Inducible Factor 1-alpha IL-6, Interleukin-6 IL-1β, Interleukin-1 beta NF-κB, Nuclear Factor kappa-light-chain-enhancer of activated B cells COX-2, Cyclooxygenase-2. This figure was created by Biorender.Com.

Succinate levels are greatly elevated in macrophages treated with LPS, in which they control HIF1α stability and thereby enhance pro-inflammatory cytokine IL-1β expression[55]. HIF-1α regulation by glycolysis within activated macrophages is critical at the inflammation site related to hypoxia. Succinate is a signaling molecule that produces expression of target genes of HIF-1 via instant ATP production and pentose phosphate pathway, increasing biosynthesis capacity of the activated cells. LPS-induced succinate inhibits directly the PHD activity in the macrophages and stabilizes HIF-1a[55] and trigger expression of a wide range of target genes. HIF-1a is also required for sustained induction of the proinflammatory cytokine IL-1b. It shows that succinate directly affects directly the HIF-1a pathway and induce macrophage response. Apart from direct inhibition, succinate indirectly stabilizes HIF-1a by inducing reactive oxygen species. Succinate dehydrogenase inhibition or RNA interference of SDH subunit B has been shown to stabilize HIF-1a in a ROS-dependent fashion[114]. Reactive oxygen species (ROS) control HIF-1α through oxidation of the Fe2+ to Fe3+, which is a critical cofactor of PHD, thereby repressing PHD activity and allowing stabilization of HIF-1α[115]. HIF-1α induces inflammation through the regulation of innate and adaptive immunity, and succinate has been shown to perform the same function as part of HIF-1α stabilization. Succinate is also an agonist of the G protein-coupled receptor (GPCR), GPR91, in inflammation. Activation of SUCNR1 is also an important component of immune cell physiology, communicating succinate, as well as HIF-1α stabilizing^[116,117]^. Succinyl-CoA is in balance with succinate within mitochondria and can modulate succinate levels when protein succinylation takes place. Protein succinylation, a newly identified PTM, therefore, plays a critical role in the metabolic switch that occurs during inflammation (Fig. 3).

### Succinylation on amino acid metabolism

The majority of cancer cells are glutamine hydrolyzed to maintain REDOX preservation, biosynthesis, and bioenergetic pathways, regulated by mitochondrial renal glutaminase GLS activity[118]. Glutaminase (Gls)-catalyzed catabolism of glutamine enhances NADPH and glutathione synthesis to combat oxidative stress[119]. Lack of GLS leads to an increase in the NADP+/NADPH ratio and a decrease in the cell GSH/GSSG ratio[120]. Consequently, GLS depletion elevates reactive oxygen species (ROS) levels. Increased hydrogen bonding between succinylated GLS’s K311 and the adjacent monomer’s H475 leads to increased activity of GLS and leads to the oligomerization of GLS. Succinylation of GLS K311 activates GLS, increasing NADPH and GSH production to fight oxidative stress ROS generation and apoptosis, tumor cell proliferation, and tumor growth[120].

### Succinylation affects the urea cycle

Ammonia is a toxic metabolite produced by amino acid metabolism in physiological conditions and catabolized by the urea cycle. Almost all of the enzymes participating in the urea cycle are masculinized in tissue of the liver[30]that affects the activity of the urea cycle. Succinylation may be responsible for the regulation of activity of these proteins and controlling the urea cycle. Glutamate dehydrogenase (GLUD1) is one of the most amylated proteins with 15 sites. GLUD1 catalyzes the glutamate oxidation deamination to a-ketoglutarate and ammonia, thereby providing a source of ammonia that feeds into the urea cycle via carbamoyl phosphate synthetase 1 (CPS1)[17]. CPS1 is the initial enzyme that is largely situated in the mitochondria, having to do with the conversion of ammonia to harmless urea in the urea cycle. There are several succinylation sites on CPS1 in mouse liver and a number of highly succinylated lysine residues on argininosuccinate synthetase 1 (ASS1), a critical enzyme of the urea cycle[121]. CPS1 has 52 acetylation sites, 39 of which are msuccinized. Succinylation of CPS1 plays a huge role in the urea cycle by controlling the conversion of ammonia and bicarbonate to carbamoyl phosphate. Conversely, ass1 succinylation at K21 can diminish the thermostability of its enzyme and further compromise the function of the urea cycle[122]. Ornithine transcarboxylase (OTC) catalyzes the conversion of ornithine and carbamoyl phosphate to citrulline, which is critical in the detoxification process of ammonia. It has been demonstrated in existing studies that lysine 88 (Lys 88) of OTC is succinylated in mouse liver, which compromises the activity of OTC and results in urea cycle blockage. Therefore, protein succinylation affects urea cycle and amino acid metabolism by modulating a variety of urea cycle metabolic enzymes.

### Transcriptional regulation of genes by succinylation

Succinylation is present everywhere in the nucleus and cytoplasm. It occurs in the cytoplasm but is primarily localized in mitochondria, where it has an important role in regulating tricarboxylic acid cycle, amino acid catabolism, and fatty acid metabolism^[54,123,124]^. Lysine succinylation is present in more than one-third of nucleosomes in the nucleus, and the locations of succinylation are primarily restricted to the promoter regions of genes. It is considered that chromatin succinylation will be one mechanism by which metabolic processes regulate transcription and do DNA repair processes genome-wide^[54,124]^. Succinylation of lysine residues in chromatin is speculated to be involved in the regulation of transcription, with modification of gene promoters able to add additional regulatory information. By making the positively charged lysine side chains negative, succinylation can bring about electrostatic changes that can promote transcription by inducing changes in chromatin. It was revealed by research that the higher the level of succinylation in the area around the gene expression and transcription initiation, the higher the gene expression[54], and also reveal that promoter succinylation can perfectly predict high gene expression. Histone succinylation destabilizes chromosomes and nucleosomes by reducing histone affinity for DNA. It is known that freeing DNA from bound protein increases the interaction of transcription factor and DNA. Succinylation at H3K122 results in promoting histone DNA interactions Reducing H3K122 succinylation enhances transcription. So, high protein succinylation promotes the transcription of genes.

Depletion of SIRT7 compromises chromatin compaction and DNA repair[125]. SIRT7 also has the activity of a dessuccinylase to be involved in the control of histone succinylation. Over-succinylation of the chromatin will also inhibit SIRT7 activity, preventing DNA repair function^[54,125]^.

Human flap endonuclease 1 (FEN1) is a key structure-specific endonuclease in DNA replication and repair maintenance. FEN1 is post-translationally modified in diverse ways, such as methylation, phosphorylation, and SUMO-1 conjugation, in DNA replication and DNA repair[126]. FEN1 possesses flap endonuclease (FEN) activity, 5ʹ-exonuclease activity, and gap endonuclease (GEN) activity^[127,128]^. GEN activity is important for DNA cleavage during apoptosis and unwinding of homologous recombination intermediates[128]. FEN1 also binds to DNA repair proteins, including the Rad9-Rad1-Hus1 complex, and participates in DNA repair activities, including the resolution of stalled DNA replication forks. FEN1 is also conjugated with SUMO-1. K200 succinylation is an important FEN1 post-translational modification, influencing protein-protein interactions and regulating other modifications, including phosphorylation and SUMO-1 conjugation[129]. FEN1 succinylation is cell cycle-regulated and DNA damage-induced in S-phase. K200 is a significant target of FEN1 succinylation, and heightened GEN activity is via augmented association with Hus1, a subunit of the Rad9-Rad1-Hus1 complex. Succinylation at K200 stimulates GEN activity as well as induces SUMO-1 modification to facilitate Rad1 and Hus1 interaction with DNA damage. Further, FEN1 succinylation enhances SUMO-1 modification via phosphorylation, also implicated in the repair of stalled DNA replication forks[129]. Thus, succinylation of FEN1 is essential to regulate its activity in DNA replication and repair for genome stability.

## Downstream effects and tumor progression

**Figure 4.**
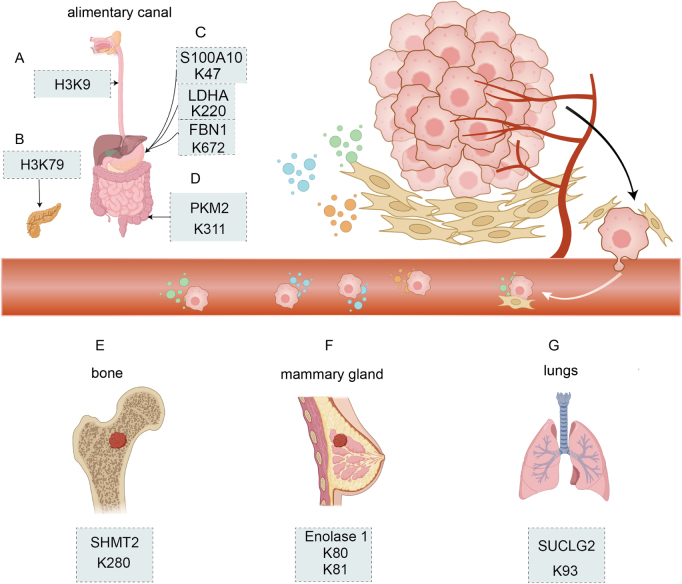
(A) H3K9 modification inhibits the proliferation of esophageal squamous cell carcinoma by decreasing levels of histone methylation. (B) H3K79 modification in pancreas activation stimulates the expression of oncogenes and stimulates the proliferation of pancreatic ductal adenocarcinoma. (C) S100A10 K47, LDHA K220, and FBN1 GI tract K672 labeling and other protein modification sites stimulate the proliferation of gastric cancer cells. (D) PKM2 K311 modification in intestinal tissue. It promotes the interaction between cancer cells and the microenvironment to produce signaling molecules and promote colon inflammation, which promotes colon cancer growth. (E) SHMT2 modification promotes osteosarcoma growth, (F) Enolase1 K80 K81 modification promotes breast cancer growth, and (G) SUCLG2 modification promotes lung adenocarcinoma growth. In (E)–(G), SHMT2 K280, Enolase 1 K80/K81, and SUCLG2 K93, tissue-specific modifications in bone, breast, and lung, are also reported, taking into account their functions, as possible metastasis bias in organs. H3K9, Histone H3 lysine 9 H3K79, Histone H3 lysine 79 S100A10, S100 calcium-binding protein A10 LDHA, Lactate dehydrogenase A FBN1, Fibrillin-1 PKM2, Pyruvate kinase M2 SHMT2, Serine hydroxymethyltransferase 2 Enolase 1, Alpha-enolase SUCLG2, Succinate-CoA ligase GDP-forming subunit beta. This image was created by Biorender.Com.

Succinylation is involved in the regulation of numerous biological processes by modification of protease function and gene transcription. Dysregulation of succinylation may be a contributor to the pathogenesis of numerous diseases, including cancers, cardiometabolic diseases, hepatic metabolic diseases, and neurological diseases. Regulators of succinylation were found to promote or inhibit cancer development by modulating the extent of succinylation of target substrates[130] (Fig. 4).

Protein lysine succinylation was found to affect tumor progression through several molecular mechanisms. It controls neoplastic cell proliferation and metastasis potential. It also plays a role in DNA damage response (DDR) and antitumor immunity. KAT2A is an essential mediator of histone succinylation and hence contributes to epigenetic gene expression[48]. KAT2A catalyzes histone H3 succinylation at Lysine 79 to enable it to bind to the Alpha-ketoglutarate dehydrogenase (alpha-KGDH) complex to receive succinyl-CoA, enhancing histone succinylation. KAT2A and the alpha-KGDH complex are both tightly linked to gene expression and tumor cell proliferation. Histone acetyltransferase 1 (HAT1) was found to regulate succinylation of histone and non-histone proteins. On the one hand, HAT1 succinylates H3 at K122 to enhance gene expression in tumor cells; on the other hand, it can accelerate its succinylation, increasing its activity and thus tumor formation^[48,131]^.

### Effect of succinylation on gastric cancer

S100A10 is highly expressed in gastric carcinoma tissues and overexpressed in metastatic lymph nodes. Based on mass spectrometry analysis, 503 sites succinylate lysine on 303 proteins were identified. S100 proteins are cytoplasmic calcium binding proteins associated with intracellular and extracellular functions. They play an important role in tumor growth, invasion, neovascularization, and metastasis^[132,133]^. S100A10 is site specific succinylated at lysine 47 by CPT1A, which results in high levels of protein expression in gastric cancer. Succinylation at K47 reduces S100A10 from its ubiquitination and proteasomal degradation and allows for plasminogen activation and gastric cancer cells[132] to migrate and invade the gastric cancer cells[132].

Lactate dehydrogenase A (LDHA) is an aerobic glycolysis rate restrictor, catalyzing the reversible interconversion between pyruvate and lactate and NADH and NAD+, providing energy for cancer cells[17]. SQSTM1 (p62) is a traditional autophagy receptor to clear proteins and pathogens, and overexpressed in most tumors[134]. K63 ubiquitinated LDHA binds to SQSTM1 to degrade it by the lysosomal pathway. LDHA succinylation at K222 is catalyzed by KAT2A and CPT1A. Experimental evidence confirms that LDHA is significantly succinylated at K222, influencing its binding activity and protein-protein interactions, which affects its structure and function. This modification diminishes the association of ubiquitinated LDHA to SQSTM1, leading to reduced degradation of LDHA. Abnormal LDHA accumulation promotes the Warburg effect in cancer cells, and overexpression also disturbs the regulation of matrix proteins, influencing gastric cancer development and progression[135]. Consequently, LDHA overexpression facilitates gastric cancer cell proliferation and migration, exerting detrimental effects on patients with gastric cancer[46].

Extracellular matrix (ECM) plays a crucial role in the regulation of gastric cancer (GC) progression. Fibrin 1 (FBN1) is a crucial component of microfibrils, extracellular matrix skeleton, inelastic and elastic tissue extracellular matrix[80]. High FBN1 levels are inversely correlated with the overall survival of GC patients. Matrix metalloproteinase-2 (MMP2) is an extracellular matrix protein involved in gastric cancer development[136]. As an extracellular matrix functional protein, MMP2 protein expression is high and degrades collagen tissue and intercellular matrix, forming a channel for invasion and infiltration of cancer cells and promoting tumor cell invasion of the basement membrane[137].Studies have shown positive correlation between MMP2 and FBN1 upregulation[138]. FBN1 is highly succinylated at K672 and K799 in GC. FBN1 is degraded by the family of MMPs, and the succinylated FBN1 inhibits its degradation by matrix metalloproteinases (MMPs). The succinylated amber group of FBN1 inhibits its binding with MMP2 and blocks degradation by MMP2, leading to FBN1 buildup. MMP2 overexpression is from transcription of HIF-1[139], and FBN1 buildup can increase the level of HIF-1 and induce MMP2 expression, leading to tumor cell invasion into matrix[138]. FBN1 is closely related to the PI3K/Akt signaling pathway in gastric cancer (GC). TGF-β1 regulates immune cells and may activate a variety of tumor-associated signaling pathways, including the MAPK and PI3K/Akt signaling cascades^[140,141]^. Succinylation of FBN1 results in its buildup, and it is able to bind with the latent complex of TGF-β1 and transmit signals by focal adhesion kinase adaptor proteins. The PI3K/Akt pathway is triggered directly in this manner[138]. FBN1 deposition and accumulation chronically with high succinylation in gastric cancer promotes tumor development through the activation of TGF-β1 and intracellular PI3K/Akt pathways, leading to a poor prognosis.

**Figure 5.**
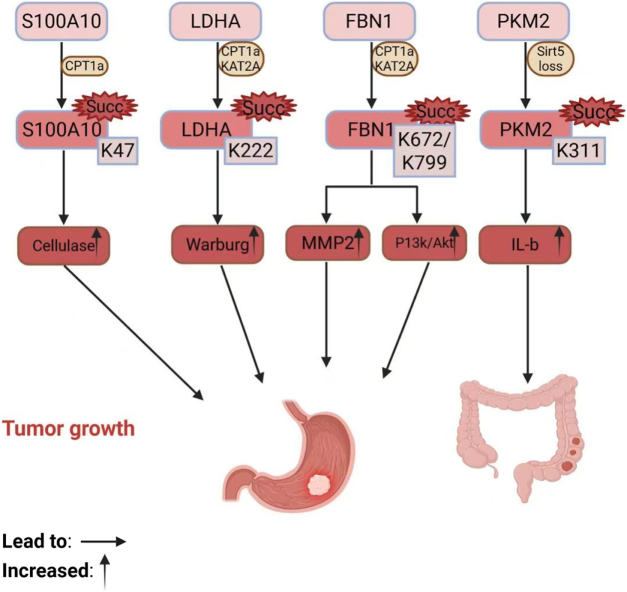
Succinylation of S100A10 at K47 not only stabilizes it but also promotes the proliferation and migration of gastric cancer cells by promoting the activation of plasminogen. KAT2A and CPT1A may enhance the succinylation of LDHA at K222, enhancing the Warburg effect in tumor cells. In addition, KAT2A and CPT1A controlled the over-succinylation of FBN1 on K672 and K799 in gastric cancer, not only breaking intercellular matrix and collagen structure but also causing tumor cells to invade the basement membrane. Concurrently, FBN1 succinylation modification leads to its accumulation and directly activates the PI3K/Akt pathway as well as promotes tumor growth. SIRT5 inhibits macrophage proinflammatory response through modifying the succinylation, activity, and function of PKM2, thereby affecting colon cancer progression. S100A10, S100 Calcium Binding Protein A10 KAT2A, Lysine Acetyltransferase 2A CPT1A, Carnitine Palmitoyltransferase 1A LDHA, Lactate Dehydrogenase A FBN1, Fibrillin-1 PI3K, Phosphoinositide 3-Kinase Akt, Protein Kinase B SIRT5, Sirtuin 5 PKM2, Pyruvate Kinase M2. This figure was created by Biorender.Com.

These results suggest that succinylation at S100A10 K47, LDHA K222, and FBN1 K672 contributes to the progression of gastric cancer (Fig. 5).

### Effect of succinylation on colon carcinogenesis

Inflammatory responses are dependent on the metabolic reprogramming of immune cells from the resting to the activated state, recapitulating the Warburg-like metabolic shift of cancer cells[142]. In macrophages stimulated with LPS, HIF1a is induced alongside a strong increase in glycolytic flux that supplies required biosynthetic precursors for the synthesis of proinflammatory proteins^[143,144]^. HIF1α is the main regulator of cell metabolism and the primary modulator of immune cell function. PKM2 was also demonstrated to act as a coactivator of phd3 activation of HIF1 and to cause transcription of HIF1-dependent enzymes required for the Warburg effect in cancer cells[145]. The enzymatic activity of PKM2 was shown to be regulated by succinylation[143]. PKM2 exists in homotetramer and homodimer structures. The dimeric PKM2 has low enzyme activity, whereas the tetrameric PKM2 is more active at physiological concentrations of phosphoenolpyruvate (PEP)[146]. Succinylation at K311 of PKM2, one of the seven identified lysine residues, favors the transition from tetrameric to dimeric form and its nuclear translocation. Unlike the tetramer, which is highly active in glycolysis, the dimeric PKM2 in the nucleus is less enzymatically active. Highly succinylated PKM2 exists mainly in an inactive monomer/dimer form[147]. In the DSS-induced colitis model of mice, after PKM2 succinylation, PKM2 translocated into the nucleus, formed a transcription complex with HIF1a, and directly bound to the IL-1b promoter gene and commenced its transcription. Therefore, high succinylation of PKM2 reduces its pyruvate kinase activity yet increases its protein kinase activity, thereby regulating the secretion of inflammatory factor IL-1b and playing a role in DSS-induced colitis in mice[147]. SIRT5 acts as a desuccinylase to regulate PKM2 activity and function[148]. High succinylation regulated by SIRT5 inhibits a pyruvate kinase activity of PKM2 by promoting the tetramer to dimer transition of PKM2 and promotes IL-1b production in lipopolysaccharide (LPS)-activated macrophages to promote inflammatory response[147]. Therefore, PKM succinylation at K311 can inhibit pyruvate kinase activity and promote IL-1b secretion, affecting colon carcinogenesis. This process is regulated by SIRT5, which suppresses the process of macrophage proinflammatory response at least in part by regulating PKM2 succinylation, activity, and function.

### Effect of succinylation on breast cancer

Succinylation of amino acid has a regulatory role in energy metabolism, and the major pathway for glucose catabolism is the pentose phosphate pathway (PPP). PPP-related protein succinylation is exceptionally high in advanced cancers, and lysine succinylation is responsible for explaining the altered patterns of protein expression. Besides, the TCA cycle produces and consumes succinate, and the PPP consumes glycolytic intermediates to produce NADPH, a chief participant of oxidative stress^[149,150]^. Thus, in activated glucose metabolism in breast cancer, the balance of succinate production and consumption determines the dynamics and patterns of succinylation and protein expression and consequently influences breast cancer (BC) formation[149]. Lysine succinylation is involved in breast cancer cell proliferation and DNA damage response (DDR) in malignancy. Numerous breast cancer-related proteins have elevated succinylation levels, particularly histone H2A, which plays a crucial role in the regulation of tumor formation. Moreover, the X complex and nucleolipin 1 (NPM1) may also be core regulators in the process[151]. X complex has the capacity to generate dynamic damage to DNA break sites under a variety of conditions of DNA damage[152]. H2A is adaptable to DNA damage stimulation and recruit many other proteins by the X complex to generate protein foci and allow for the repair of DNA damage[153]. The process could be regulated by protein modification. Thus, hyperacetylation and succinylation of H2A protein play a role in the repair of DNA and the formation of a tumor. Thus, histone succinylation can regulate gene transcription and consequently influence cell proliferation and tumor formation[149].

Carnitine palmitoyltransferase 1A (CPT1A) has lysine succinyltransferase (LSTase) activity in both biological and in vitro conditions. Thus, CPT1A regulates cell metabolism by substrate protein succinylation[154]. Enolase is one of the most highly succinized proteins mediated through CPT1A[73]. CPT1A can succinylation enolase 1 and damage enolase’s enzymatic activity both in cells and in vitro[155]. Experiments proved that CPT1A acts as an LSTase to inhibit enolase 1 enzyme activity and promote the proliferation of breast cancer cells under glutamine starvation. Enolase 1 activity was inhibited by succinylation of enolase 1 at K80/81 and K335 by CPT1A. Inhibition of enolase 1 expression can induce senescence in breast cancer cells but can support cell survival when glutamine metabolism is inhibited by the glutaminase inhibitor BPTES(bis-2-[5 phenylacetamino-1,2, 4-thiadiazole-2-yl] ethyl sulfide)[156]. Glutamine is therefore predominantly used in breast cancer cells. CPT1A facilitates glutamine-independent growth of breast cancer cells by increasing the global lysine succinylation status in cells, especially the succinylation of enolase 1 at K80/81 and K335, which inhibits enolase activity.

### Effect of succinylation on esophageal squamous cell carcinoma

Elevated levels of lysine succinylation have been associated with enhanced tumor proliferation and metastasis in certain cancer types, while exerting inhibitory effects on neoplastic progression in others. Notably, treatment with dimethyl succinate (DMS) promotes global succinylation in esophageal adenocarcinoma cells but suppresses this post-translational modification in esophageal squamous cell carcinoma (ESCC)[157]. Succinylation of lysines in TCA cycle protein lysines was notably lower in ESCC cells at the level of PTM. Lysine succinylation may inhibit the activity of its protein substrates, such as phosphoenolpyruvate carboxylate kinase 2 (PCK2), aconitate hydrated hemitochondria (ACO2), and fumarate hydratase (FH)[157], which regulate tumor phenotypes. The migratory and proliferative behaviors of ESCC cells may be attenuated by this modification. Meanwhile, histone lysine methylation has been shown to facilitate the invasive capacity of ESCC cells[158]. High-level succinylation regulates and inhibits histone methylation^[94,157]^. An elevated level of succinylation at histone H3K9 has been linked to a decrease in histone methylation. Accordingly, enhancing lysine succinylation at specific residues in ESCC cells may contribute to the inhibition of their proliferation and migratory potential.

### Effect of succinylation on lung adenocarcinoma

Oncogenesis usually occurs with dysfunctional mitochondrial metabolism and metabolic processes^[159–161]^. Previous studies have indicated that SUCLG2 expression influences both the proliferative capacity and invasive behavior of lung adenocarcinoma cells[162]. SUCLG2 is the major hydrolase of succinyl-CoA. Succinyl-CoA is the master succinylation donor, and its level is regulated by succinyl-CoA synthetase (SUCL)[25]. SUCLG2 expression is markedly upregulated in lung adenocarcinoma (LUAD) tissues relative to adjacent normal counterparts. Given its essential role in maintaining mitochondrial homeostasis, elevated SUCLG2 levels have been strongly correlated with poor patient prognosis. Succinylation at lysine 93 (K93) enhances the stability of the SUCLG2 protein, thereby facilitating LUAD cell proliferation and tumor progression[25]. In contrast, loss of SUCLG2 increases the succinylation levels of mitochondrial proteins, which may compromise mitochondrial function in lung adenocarcinoma cells by diminishing enzymatic activity[163]. SIRT5, a mitochondrial sirtuin, directly interacts with SUCLG2 and removes the succinyl group at lysine 93 (K93). This de-succinylation event results in reduced protein stability of SUCLG2. Following the loss of succinylation mediated by SIRT5, the E3 ubiquitin ligase (TRIM21) targets SUCLG2 at lysine 200 (K200) through K63-linked ubiquitination, thereby facilitating the recruitment of the p62 adaptor protein and directing SUCLG2 toward lysosomal degradation^[132,163]^. While this pathway is functional in normal cells, it is suppressed in lung adenocarcinoma cells due to reduced TRIM21 activity or expression, leading to SUCLG2 succinylation, increased stability, abnormal accumulation, and enhanced tumor cell proliferation. Thus, SUCLG2 regulates mitochondrial protein succinylation in a dual manner: on one hand, by hydrolyzing succinyl-CoA, it reduces succinylation levels and maintains the activity of key metabolic enzymes; on the other hand, its own succinylation enhances protein stability via the SIRT5-TRIM21 axis, forming a cancer-promoting positive feedback loop. These insights highlight the succinylation pathway as a promising therapeutic target in lung adenocarcinoma.

### Effect of succinylation on pancreatic ductal adenocarcinoma

Histone modifications modulate chromatin architecture and influence DNA-histone interactions, thereby controlling the binding accessibility of histone-associated proteins. These modifications critically govern all chromatin-templated biological processes[164]. Among the recently discovered post-translational modifications of human histones, lysine succinylation occurs frequently^[2,30]^. Recently, it was found that the histone acetyltransferase (HAT) KAT2A(also known as GCN5) can act as succinyltransferase[48], which can regulate the succinylation of histone H3 affecting gene expression leading to the deterioration of pancreatic ductal adenocarcinoma (PDAC). High expression of β-catenin can promote glycolysis, cell proliferation, migration, and invasion of PDAC cells, and epithelial-mesenchymal transition. The stability of β-catenin is enhanced through its interaction with 14-3-3ζ (encoded by YWHAZ), which mediates β-catenin degradation and promotes downstream transcriptional activity^[165,166]^. It was found that the succinylation of histone H3 mediated by KAT2A occurs at lysine K79 with the highest frequency near the transcription start site of the gene. Therefore, it was proved that KAT2A enhanced 14-3-3ζ expression by regulating H3K79 succinylation in the YWHAZ promoter region (encoding 14-3-3ζ)[74]. Meanwhile, overexpression of 14-3-3ζ disrupted the interaction between β-catenin and β-TrCP E3 ligase, upregulated the expression of β-catenin, and promoted PDAC epithelial-mesenchymal transition[74]. These findings revealed that KAT2A modulates the succinylation of histone H3 on K79 and subsequent gene transcription[48]. It was shown that histone succinylation is essential to regulating gene expression and β-catenin stability as well as PDAC cell proliferation and invasion.

### Effect of succinylation on osteosarcoma

Mitochondrial SHMT2 controls the rate-limiting step of the serine catabolic pathway to facilitate cancer cell growth. Notably, SHMT2 is a hypoxia-inducible effector that is transcriptionally upregulated to support cell survival in hypoxia[167], and its gene expression by enhancing glioma cell survival during ischemia[168]. SHMT2 is a regulatory node that coordinates serine catabolism and one-carbon metabolism, where small molecule metabolites have a central role in the regulation of cell proliferation and REDOX homeostasis. Serine has a strong function in one-carbon metabolism and oncogenesis, and serine is the only amino acid that acts as an activator of PKM2, while one-carbon metabolism driven by serine has been recognized as an important pathway for the generation of NADPH^[169,170]^. Existing research has established that in osteosarcoma cell lines, succinylation at SHMT2-K280 causes the protein SHMT2 to form an inactive dimeric structure to suppress enzymatic function. The widespread succinylation of SHMT2 limits the serine to glycine metabolic reaction that proceeds through subsequent one-carbon metabolism, inhibiting cellular REDOX equilibrium and clonal cell growth[171]. Therefore, succinylation of SHMT2 in osteosarcoma cells other than ESCC inhibits osteosarcoma cell growth by suppressing SHMT2 enzymatic activity and metabolic flow. Meanwhile, SHMT2 K280 succinylation is regulated by SIRT5, and the post-translational modification is inversely linked with osteosarcoma cell proliferation and tumor growth via regulation of the mitochondrion-carbon metabolism pathway (Table 2).

**Table 2:** Expression, gene symbol, influence, site, and regulatory actors of succinylation in tumors

Tumor	Gene symbol	Impact in tumors	Ksucc sites	Regulatory factors of succinylation	References
Gastric cancer	S100A10	Promote tumor growth and migration	K47	CPT1A	[132]
GC	FBN1	Degradation of extracellular matrix	K672/K799	CPT1A/KAT2A	[138]
		Promote tumor invasion of the basement membrane			
GC	LDHA	Promote the growth and metastasis of gastric cancer	K222	CPT1A/KAT2A	[80]
Breast cancer	PKM2	Inhibit cell proliferation and tumor growth	K498	sirt5	[172]
BC	Enolase1	Enhanced non-dependent BC cell proliferation	K80/81/K335	CPT1A	[149]
ESCC	H3	Inhibit ESCC growth and migration	K9	sirt5	[157]
LUAD	SUCLG2	Promote the proliferation of tumor cells	K93	sirt5	[25]
PDAC	H3	Regulate gene expression and promote PDAC epithelial interstitialization	K79	sirt5	[74]
Colon cancer	PKM2	Regulation of macrophages in malignant transformation of colon cancer	K311	sirt5	[147]
Osteosarcoma	SHMT2	Reduce enzyme activity and inhibit the growth of osteosarcoma	K280	sirt5	[171]

## Targeted therapy strategies and future research directions

PTMs are involved in important biological processes, and disease development and progression are regulated by various PTMs[173]. Recent progress in tumor immunotherapy has revealed that succinylation plays a critical role not only in regulating intracellular metabolic pathways but also in profoundly influencing immune cell proliferation, activation, and metabolic reprogramming within the tumor microenvironment^[27,100]^. Accordingly, therapeutic strategies targeting succinylation may offer novel opportunities for the treatment of various diseases, particularly malignancies.

### Regulation of succinylation and nervous system diseases: from molecular mechanisms to precision treatment strategies

Succinylation modification exhibits significant biological effects in a variety of neurodegenerative diseases and metabolic disorders[174]. Studies have shown that other psychiatric disorders, such as schizophrenia, and stroke are strongly associated with disturbances of mitochondrial function and metabolism^[28,175,176]^. Antipsychotic drugs that modulate succinylated post-translational modifications to improve symptoms include chlorpromazine, haloperidol, and quetiapine^[175]^. Antipsychotic drugs improve metabolic disorders and neurological dysfunction by inhibiting succinylation in pathways such as RNA metabolism, protein translation, and cellular stress responses, a mechanism that is closely related to their clinical efficacy^[177–180]^. Similarly, lysine succinylation plays a key role in mitochondrial energy metabolism after ischemia during stroke pathology^[176,181]^, and SIRT5 acts as a mitochondrially localized NAD+ dependent lysine deacylase that regulates protein succinylation levels and maintains mitochondrial metabolic homeostasis^[106,182,183]^. Enhanced SIRT5 desuccinylation activity was shown to have therapeutic potential in alleviating ischemica-caused mitochondrial damage, resulting in the relief of stroke symptoms[19]. Therefore, the identification of SIRT5 small molecule agonists, including resveratrol derivatives BML-217 and MC3138^[184,185]^, presents an effective therapeutic way to treat mitochondrial metabolic diseases and to relieve nerve injury. Nevertheless, further efforts are still necessary in the future to construct highly selective SIRT5 agonists with favorable pharmacokinetics and further to reveal its therapeutic benefit and mechanism in central nervous system disorders. In parallel, it is important to construct a succinylation substrate proteomic database and chart disease-specific succinylation targets, to create patient-specific succinylation profile based precision medicine strategy, and realize the transition from basic research to clinical individualized therapy. These studies will unveil novel molecular targets and individualized therapeutic strategies for mental diseases, metabolic diseases, and stroke, and enable clinical translation of succinylation modification in medicine.

### From fungal infection to tumor microenvironment: therapeutic potential and research prospects of succinylation regulation

After the emergence of drug-resistant strains, the therapeutic mortality rate of Aspergillus fumigatus-infected patients is also high[186]. Experiments have shown that there are large differences in sites of succinylation among strains with different itraconazole (ITR) resistances[187], and the deaminating enzyme blocker nicotinamide (NAM) has a synergistic bactericidal effect and enhances macrophage killing activity against ITR-resistant Aspergillus fumigatus[188]. The above findings for antifungal drug resistance treatment present a new idea. Highly cellular metabolism- and hypoxia-sensitive post-translational modification, succinylation is also a key regulator of the tumor immune microenvironment[41]. From one perspective, succinylation affects processes of tumor immune evasion by interfering with energy metabolism and immune cell metabolic reprogramming[189]. From another perspective, Succinate released by tumors polarizes macrophages to facilitate cancer metastasis^[180,190]^, as well as stimulating the antigen-presenting function of dendritic cells^[27,180]^. At the molecular level, succinylation of a specific protein, for example, CPT1A, causes succinylation of SP5 at K391, which activates the master signaling pathway PDPK1-AKT/mTOR and promotes proliferation and survival of prostate cancer cells^[6,33,191]^. Therefore, the molecular mechanism of succinylation in controlling the triazole resistance phenotype by regulation of TCA cycle, carbon and nitrogen metabolism, and other important metabolic processes is explored and the key target proteins of succinylation are identified as putative therapeutic targets. In contrast, study the synergistic effect of combined application of NAM and other desuccinase inhibitors with existing antifungal drugs and their impact on fungal metabolic remodeling, to provide a theoretical basis and clinical transformation basis for novel antifungal treatment based on succinylation regulation. Though succinylation is regulated by enzymatic and nonenzymatic mechanisms, their regulators also have direct and indirect effects on tumor growth[70]. Further research on the key enzymes regulating the level of succinylation (Sirtuin 5, CPT1A.) as drug targets is of extreme significance for increasing the survival time and state of cancer patients.

### Therapeutic applications of protein succinylation in surgical oncology: metabolic regulation and translational perspectives

Surgery is the main radical cure for solid tumors, but the therapeutic effect is usually affected by tumor invasion and metastasis, postoperative recurrence, and immune microenvironment regulation[192]. Recent studies have shown that succinylation modification is closely related to surgical precision treatment strategy by regulating tumor metabolic reprogramming and immune escape. In the surgical treatment of invasive tumors, succinylation modification is particularly critical to the regulation of extracellular matrix (ECM) remodeling. For example, K672/K799 succinylation of FBN1 protein in gastric cancer can inhibit ECM degradation mediated by MMP2-2 and promote tumor cell invasion. Succinyltransferase inhibitors (CPT1A inhibitors) developed for this pathway can be combined with surgery to block ECM remodeling and reduce the risk of tumor cells shedding and metastasis during surgery^[138,193,194]^. In addition, markers related to succinylation (such as the expression level of SUCLG2) may be used as molecular indicators to evaluate the prognosis of surgery. The high succinylation of SUCLG2 in lung adenocarcinoma is related to mitochondrial dysfunction and low survival rate of patients. Monitoring its expression after surgery may provide a basis for individualized adjuvant therapy^[25,195]^. Succinylation is capable of modifying upstream regulatory proteins, including IKK and TBK1, which in turn augments their functional activity and subsequently promotes the activation of the NF-κB signaling cascade^[38,196]^. Activation of this pathway directly upregulates the expression of the immunosuppressive molecules PD-L1 and CD47, conferring an immune escape advantage to tumor cells and facilitating tumor growth and metastatic dissemination[197]. Accordingly, modulation of succinylation during the perioperative phase offers both a conceptual framework and a feasible strategy for precise immunomodulation, emerging as a novel molecular target to mitigate the risk of postoperative tumor recurrence and metastatic spread[198]. Based on succinylation, it can provide a new perspective for the transformation research of surgical treatment in the future: for example, the preoperative tumor metabolic classification of succinylation modified spectrum can guide the choice of surgical methods[199]; the activity of tumor cells was inhibited by succinylation regulator applied locally during operation[200]; after operation, the anti-tumor immunity was enhanced by regulating the succinylation-immune axis, and the metastasis and recurrence rate was reduced[201]. Succinylation modification, serving as a critical link between metabolic processes and immune responses, has revolutionarily transformed our understanding of surgical oncology[130]. Spanning the continuum from preoperative assessment and intraoperative intervention to postoperative surveillance and extended follow-up, succinylation offers novel insights and methodologies. Thorough investigation of these approaches is anticipated to facilitate the translation of succinylation regulation from fundamental studies to an innovative paradigm of precision surgical therapy.

## Conclusions

Lysine succinylation is a novel post-translational modification of proteins, and it has been found to be essential for regulation of tumor microenvironment and immune escape of tumors in recent years[27]. Metabolic reprogramming is the inherent mechanism of succinylation in the regulation of tumor development. Succinylation regulates the stability and activity of crucial metabolic enzymes (GLS, LDHA, PGAM1, GLUD1.) and affects metabolic pathways, as well as alters the energy metabolism status of cancer cells^[33,130]^. In addition, hypoxia, acidity, and metabolite buildup within the tumor microenvironment may affect the level of succinyl-CoA and regulate the activity of enzymes in the succinylation pathway, including SIRT5, KAT2A, and CPT1A^[74,81,202]^, constructing a dynamic network of enzymatic modification impacting tumor development. However, throughout tumor development, succinylation and associated proteins do not regulate metabolic reprogramming in a consistent manner within different types of tumors nor act comparably in tumor progression. In gastric cancer research, liver cancer, kidney cancer, colorectal cancer, and prostate cancer, succinylation manifests as inhibiting the TCA cycle and activating glycolysis, being in favor of tumor metabolic reprogramming^[6,75,203–207]^. Downregulation of succinylation in ESCC suggests that it may be a tumor suppressor[157]. In lung adenocarcinoma and studies, it can catalyze enzymes such as SDH, enhance oxidative phosphorylation, and induce ROS accumulation^[208,209]^, having an effect on mitochondrial metabolic function. At the same time, succinylation of immune regulation is able to up-regulate immunosuppressive molecules by activating pathways such as NF-κB[75] and also induce pro-inflammatory response in some immune cells with enhanced antigen presentation and inflammatory factor expression^[38,210]^.

There is also extensive cross-regulation among succinylation and other PTMs^[29,89,211–213]^. Succinylation and acetylation cooperatively regulate protein function and metabolism in lung adenocarcinoma, modulating significant signaling pathways such as ERBB and mTOR to promote tumor growth[214]. They may be redundant expressed on the same protein modification site, affecting target protein structure and conformation as well as transcriptional regulation ability, and coordinately affect cellular transcription and mitochondrial function^[29,211]^. On the level of histone modification, succinylation can coordinate with epigenetic marks such as methylation to regulate the immune-related gene expression[215]. Succinylation also promotes ubiquitination by changing the conformation of the substrate, thus regulating the protein degradation pathway and regulating the apoptosis and inflammatory response of cardiomyocytes^[208,216]^. In summary, even though currently available research on succinylation is still in its early stage, its potential for multi-level regulation of tumor metabolic reprogramming and immune evasion has emerged step by step. This review, grounded in current literature, critically examines the regulatory mechanisms governing succinylation modifications and their associated enzymes within the tumor microenvironment and provides a detailed account of recent advances in the application of succinylation in cancer therapeutics, with particular emphasis on developments in surgical oncology. In contrast to previous studies, this review highlights the central role of succinylation-related enzymes in regulating tumor metabolic reprogramming and promoting immune escape. In particular, it draws attention to the perioperative application of SIRT5 activators and CPT1A inhibitors, therapeutic approaches that demonstrate significant potential for enhancing surgical efficacy^[130,172]^. These enzymes serve as key regulators of both tumor initiation and progression, and additionally present as promising targets for adjuvant therapies in the postoperative context^[217–219]^. Succinylation modification offers a novel conceptual framework for optimizing multimodal treatment strategies and propelling the development of personalized precision medicine in the postoperative management of malignancies. Ongoing progress in translational research is driving the transition from traditional surgical paradigms to precision-guided molecular surgery, signifying a pivotal shift in the future landscape of surgical oncology. This rapidly evolving domain warrants continued attention and in-depth exploration by both clinicians and biomedical researchers.

## Data Availability

All data relevant to this review are included in the text, references, and figures.

## Research registration unique identifying number (UIN)

This study did not involve human subjects and was based on retrospective data analysis/literature review. Hence, research registration was not applicable.
